# Socioeconomic status is not associated with health-related quality of life in a group of overweight middle-aged men

**DOI:** 10.7717/peerj.5193

**Published:** 2018-07-13

**Authors:** José G.B. Derraik, Benjamin B. Albert, Martin de Bock, Éadaoin M. Butler, Paul L. Hofman, Wayne S. Cutfield

**Affiliations:** 1 Liggins Institute, University of Auckland, Auckland, New Zealand; 2 A Better Start—National Science Challenge, Auckland, New Zealand; 3 Department of Women’s and Children’s Health, Uppsala Universitet, Uppsala, Sweden; 4 Department of Paediatrics, University of Otago, Christchurch, New Zealand

**Keywords:** Male, Wealth, Socioeconomic status, Deprivation, Neighbourhood, Adult, Well-being, Health, Health-related quality of life, Residence

## Abstract

Socioeconomic status is a known determinant of health. In secondary data analyses, we assessed whether socioeconomic status affected health-related quality of life in a group of overweight (body mass index 25–30 kg/m^2^) middle-aged (45.9 ± 5.4 years) men, recruited in Auckland (New Zealand). Health-related quality of life was assessed with SF-36v2 three times: at baseline, and 12 and 30 weeks later. Socioeconomic status was determined by geo-coded deprivation scores derived from current address using the New Zealand Index of Deprivation 2006 (NZDep2006), as well as capital value of residence. Univariable and multivariable analyses showed no associations between measures of socioeconomic status and any mental or physical health domains. Our findings may reflect the fact that these men are not currently experiencing comorbidities associated with overweight.

## Introduction

Socioeconomic status (SES) is a major determinant of health. Across the socioeconomic spectrum there are stepwise improvements in mortality and morbidity with increasing wealth (Adler & Ostrove, 1999; Braveman & Gottlieb, 2014). SES is likely to affect health through complex direct and indirect pathways (Adler & Newman, 2002; Braveman & Gottlieb, 2014). For example, people with lower incomes report greater financial obstacles to effective treatment, often leading to delayed diagnosis and suboptimal management of health conditions (Osborn et al., 2016).

Health-related quality of life (HRQL) refers to the effects of health, illness and treatment on perceived quality of life (Ferrans et al., 2005). SES has previously been described to affect HRQL in a nationally representative cohort of Canadian adults (Ross et al., 2012), in adults with a chronic disease in Germany (Mielck, Vogelmann & Leidl, 2014), and among males (but not females) in Japan (Yamazaki, Fukuhara & Suzukamo, 2005). Among patients with rheumatoid arthritis and other chronic conditions, there was evidence that lower income was associated with poorer HRQL (Alishiri et al., 2008; Ovayolu, Ovayolu & Karadag, 2011), but females made up the vast majority of both study populations. Of note, in the USA, disparities in self-rated health when stratified by income group were widest in adults aged 45 to 54 years (Robert et al., 2009). Previous studies have also indicated that poorer SES is associated with adverse metabolic outcomes, but two of these studies observed this association only among women (Chichlowska et al., 2008; Lim et al., 2012), with a third investigation showing only a week association among male participants (Loucks et al., 2007).

We are not aware of any previous study in New Zealand into the association between SES and HRQL in middle-aged males. Importantly, the prevalence of obesity among adult males in New Zealand increased from 26% in 2006/07 to 30.5% in 2015/16, and it was highest in the most deprived areas at 40.7% (Ministry of Health, 2016). Thus, in light of previous evidence and given the increasing prevalence of obesity in New Zealand men (and its particularly high prevalence in areas of low socioeconomic status), we aimed to investigate whether lower SES would also be associated with HRQL in a phenotypically homogenous group of overweight middle-aged men.

## Methods

### Participants

This study consisted of secondary analyses of data from a 30-week randomized crossover trial in Auckland (New Zealand) assessing the effects of supplementation with olive leaf extract on insulin sensitivity (De Bock et al., 2013). Participants were middle-aged men (35–55 years) who were overweight (body mass index 25–29.99 kg/m^2^). Key exclusion criteria in the original trial were tobacco smoking, use of illicit drugs, diabetes mellitus, or taking any medications that could affect insulin sensitivity. In the current study, participants with incomplete HQRL data were also excluded.

### Assessments

SES was determined for each participant by geo-coded deprivation scores derived from current address using the New Zealand Index of Deprivation 2006 (NZDep2006) (Salmond, Crampton & Atkinson, 2007). This index is based on household census data reflecting nine aspects of material and social deprivation to divide New Zealand into tenths (scored 1–10) by residential address (Salmond, Crampton & Atkinson, 2007). Scores of 1 represent the least deprived areas and 10 the most deprived ones (Salmond, Crampton & Atkinson, 2007). Scores are derived from units covering a small area, each reflecting approximately 87 people (Salmond, Crampton & Atkinson, 2007). Although NZDep2006 scores apply to areas rather than individual people, they are considered reasonable indicators of SES where in depth individual measures are unavailable (Salmond & Crampton, 2012). In addition, the capital value of each participant’s residence was obtained from publicly available data from the Auckland City Council.

Each participant had HRQL assessed at the start of the trial and then at 12 and 30 weeks, in order to provide a more robust estimate of well-being for each individual. HRQL was assessed with the validated New Zealand/Australia adaptation of the SF-36v2 Health Survey (Frieling, Davis & Chiang, 2013). The SF-36v2 is based on subjective measures of well-being, measuring perception of health covering well-being, functional status, and overall evaluation of health (Ware Jr, 2000). The SF-36v2 assesses four physical health domains (bodily pain, physical functioning, role limitations related to physical problems, and general health) and four mental health domains (social functioning, vitality, role limitations related to emotional problems, and general mental health). Each individual domain is made up by a number of specific items, which have been described and discussed in detail by Ware Jr (2000). Physical and mental health domains were also quantified using summarizing scores.

Weight and height were measured during clinical assessments, and body mass index (BMI) calculated. The long format of the International Physical Activity Questionnaire (IPAQ) was used to assess physical activity levels (Hagstromer, Oja & Sjostrom, 2006). The questionnaire was self-administered and was completed during clinical assessment, reporting on the participant’s physical activity levels over the previous 7 days. The IPAQ covers four domains of physical activity: work-related, transportation, housework/gardening, and leisure time. Its validity has been shown against accelerometer data, so that it has been used widely, across a diverse range of populations.

Nutritional intake was evaluated using 3-day dietary records that were collected at each clinical visit. These records contained itemized descriptions of dietary intake during one day in the weekend and two week days (i.e., Monday to Friday). Nutritional intake was recorded using standard household measures and information obtained from food labels if necessary. A single trained investigator instructed all participants and also reviewed all records, to correct any errors or omissions, and clarify any unclear entries. The same investigator entered all records into the Foodworks software (v6.0, Xyris Software, Brisbane, Australia).

### Statistical analysis

Pearson’s correlation coefficients or Spearman’s rank correlations were initially run. Associations between NZDep2006 scores and SF-36v2 outcomes were examined using general linear mixed models based on repeated measures. Models adjusted for randomization sequence, timing of assessment, and on-going use of cholesterol-lowering and/or antihypertensive medications, as well as participant’s age, BMI, and physical activity level (IPAQ score).

Apart from continuous associations, stratified analyses were performed splitting the group in half according to the levels of socioeconomic deprivation: Lower SES (NZDep2006 scores 4 to 10) and Higher SES (scores 1 to 3). Baseline differences between groups were examined using t-tests, Chi-square tests, or Fisher’s exact tests, as appropriate.

Identical continuous and stratified analyses were also run for capital value of residence. The two groups for the stratified analyses were: Lower capital value (NZ $230,000 to 590,000) and Higher capital value (≥NZ $600,000).

Statistical analyses were performed using Minitab v.16 (Pennsylvania State University, State College, PA, USA) and SAS v.9.3 (SAS Institute Inc. Cary, NC, USA). Where appropriate, outcomes were log-transformed to approximate a normal distribution prior to analyses. Outcome data are presented as estimated marginal means adjusted for the confounding factors in multivariable models with respective 95% confidence intervals (back-transformed for logged data). All tests were two-sided, with statistical significance maintained at *p* < 0.05, without adjustments for multiple comparisons.

### Ethics

The original trial was registered with the Australian New Zealand Clinical Trials Registry (#336317), with ethics approval granted by the Northern Y Regional Ethics Committee (NTY/11/02/015). All participants provided written informed consent.

## Results

Forty-five subjects participated in the clinical trial (De Bock et al., 2013), but seven had incomplete HQRL data and were excluded. Thus, we studied 38 overweight men (BMI 27.3 ±1.4 kg/m^2^) aged 45.9 ±5.4 years (range 34.5–55.6 years), who were mostly of New Zealand European ethnicity (89%). Three participants were on antihypertensive medication, three were on lipid-lowering medications, and two participants were on both. No participants had any other physical or mental health co-morbidities.

Comparisons between our group of participants and New Zealand normative data have been previously reported (Derraik et al., 2014). Briefly, our cohort had similar scores in all mental health domains when compared to normative data, but our participants displayed better role physical (*p* < 0.001), physical functioning (*p* < 0.001), bodily pain (*p* = 0.012), and general health (*p* = 0.009) scores (Derraik et al., 2014).

### NZDep2006

NZDep2006 scores were not correlated with participants’ scores in any mental or physical health domain. Multivariate models examining linear associations also yielded negative results. Subsequent stratified analyses corroborated these negative findings. Participants of lower SES displayed similar scores across all mental and physical health domains as participants of a higher SES (Table 1). This lack of association was observed even when models adjusted for important confounding factors (Table 1). Note that there were also no differences in demography, clinical parameters, dietary intake, or physical activity levels between SES groups (Table 1).

**Table 1:** Health-related quality of life (HRQL) data on 38 middle-aged overweight men according to socioeconomic status as per NZDep2006 scores. Each participant was evaluated three times over a 30-week period. Baseline data are means ± standard deviations or *n*(%). Physical health and mental health data are estimated marginal means and respective 95% confidence intervals from general linear mixed models based on repeated measures, adjusted for randomization sequence, timing of assessment, and on-going use of cholesterol-lowering and/or antihypertensive medications, as well as participant’s age, BMI, and physical activity level (IPAQ score). Lifestyle data are estimated marginal means and respective 95% confidence intervals from general linear mixed models based on repeated measures. Note that higher physical and mental health scores represent better outcomes; lower NZDep2006 indicate lower levels of socioeconomic deprivation (i.e., wealthier status).

		Lower socioeconomic status	Higher socioeconomic status	*p*-value
***n***		18	20	
**Baseline demography**	NZDep2006	6.2 ± 2.1	2.4 ± 0.8	<0.0001
	Capital value of residence (NZ$)	565,313 ± 164,747	782,368 ± 353,767	0.025
	Age (years)	45.1 ± 5.7	46.7 ± 5.2	0.37
**Baseline clinical data**	BMI (kg/m^2^)	27.4 ± 1.5	27.6 ± 1.3	0.71
	Taking cholesterol-lowering medication	4 (22%)	1 (5%)	0.17
	Taking antihypertensive medication	4 (22%)	1 (5%)	0.17
	Systolic blood pressure (mmHg)	125.2 ± 8.4	124.5 ± 11.6	0.84
	Diastolic blood pressure (mmHg)	78.4 ± 6.0	78.1 ± 7.3	0.88
**Lifestyle**	Physical activity levels (IPAQ score)	2,117 (1,329–3,372)	1,902 (1,226–2,951)	0.73
	Total energy intake per day (kJ)	9,428 (8,576–10,279)	9,222 (8,381–10,064)	0.73
	Energy from saturated fat (%)	13.2 (12.2–14.2)	12.5 (11.5–13.5)	0.31
	Energy from sugar (%)	15.5 (13.5–17.5)	16.9 (14.9–18.9)	0.29
	Fibre intake per day (g)	23.3 (20.4–26.7)	23.3 (20.3–26.6)	0.98
	Consumed any alcohol	16 (89%)	17 (85%)	0.99
	Energy from alcohol (%)	6.5 (4.1–8.9)	5.5 (3.1–7.8)	0.53
**HRQL**	**Physical health**			
	Physical component summary	56.4 (54.9–57.9)	57.4 (55.5–59.3)	0.32
	General health	76.1 (71.0–81.3)	78.3 (72.1–84.5)	0.56
	Physical functioning	95.4 (92.4–98.5)	93.3 (89.6–97.0)	0.37
	Role limitations due to physical problems	96.0 (93.6–98.5)	95.0 (92.1–98.0)	0.58
	Bodily pain	80.6 (74.9–86.4)	82.5 (75.5–89.4)	0.66
	**Mental health**			
	Mental component summary	52.2 (49.3–55.1)	50.3 (46.8–53.8)	0.36
	Mental health	78.3 (73.3–83.4)	75.1 (69.0–81.3)	0.38
	Vitality	65.8 (60.1–71.6)	63.4 (56.4–70.3)	0.55
	Social functioning	92.0 (85.2–98.7)	90.1 (81.9–98.3)	0.70
	Role limitations due to emotional problems	92.2 (87.2–97.1)	88.6 (52.6–94.6)	0.32

### Capital value of residence

Information on capital value was available for 35 of 38 participants. Men living in residences of a higher capital value were 4.2 years older on average (*p* = 0.020), but had similar clinical characteristics at baseline, physical activity levels, and dietary intake (Table 2). Similarly to NZDep2006, there were no significant correlations between residential capital value and any mental or physical health domains, with multivariate models providing similar results. Stratified analyses yielded no significant differences in physical or mental health domains between groups separated according to capital value (Table 2).

**Table 2:** Health-related quality of life (HRQL) data on 35 middle-aged overweight men according to the capital value of their residential address. Each participant was evaluated three times over a 30-week period. Baseline data are means ± standard deviations or *n*(%). Physical health and mental health data are estimated marginal means and respective 95% confidence intervals from general linear mixed models based on repeated measures, adjusted for randomization sequence, timing of assessment, and on-going use of cholesterol-lowering and/or antihypertensive medications, as well as participant’s age, BMI, and physical activity level (IPAQ score). Lifestyle data are estimated marginal means and respective 95% confidence intervals from general linear mixed models based on repeated measures. Note that higher physical and mental health scores represent better outcomes; lower NZDep2006 indicate lower levels of socioeconomic deprivation (i.e., wealthier status).

		Lower capital value	Higher capital value	*p*-value
***n***		17	18	
**Baseline demography**	Capital value of residence (NZ$)	450,882 ± 93,261	902,500 ± 259,718	<0.0001
	NZDep2006	4.7 ± 2.9	3.6 ± 1.9	0.20
	Age (years)	43.6 ± 5.0	47.8 ± 5.3	0.020
**Baseline clinical data**	BMI (kg/m^2^)	27.7 ± 1.4	27.4 ± 1.4	0.58
	Taking cholesterol-lowering medication	2 (12%)	3 (17%)	0.99
	Taking antihypertensive medication	2 (12%)	3 (17%)	0.99
	Systolic blood pressure (mmHg)	124.8 ± 8.0	125.8 ± 12.4	0.78
	Diastolic blood pressure (mmHg)	78.6 ± 6.1	78.9 ± 7.0	0.88
**Lifestyle**	Physical activity levels (IPAQ score)	2,216 (1,373–3,577)	1,866 (1,174–2,964)	0.60
	Total energy intake per day (kJ)	8,756 (8,073–9,439)	9,243 (8,532–9,954)	0.32
	Energy from saturated fat (%)	13.6 (12.6–14.6)	12.4 (11.4–13.4)	0.10
	Energy from sugar (%)	15.9 (13.7–18.0)	16.5 (14.3–18.7)	0.65
	Fibre intake per day (g)	22.8 (19.9–26.2)	22.8 (19.8–26.3)	0.99
	Consumed any alcohol	14 (82%)	16 (89%)	0.99
	Energy from alcohol (%)	5.7 (3.2–8.3)	5.8 (3.4–8.1)	0.99
**HRQL**	**Physical health**			
	Physical component summary	57.7 (56.0–59.4)	55.8 (54.1–57.5)	0.06
	General health	77.0 (70.9–83.0)	76.6 (70.7–82.6)	0.93
	Physical functioning	95.0 (91.3–98.7)	94.3 (90.7–97.9)	0.83
	Role limitations due to physical problems	96.9 (94.1–99.7)	94.7 (92.0–97.3)	0.22
	Bodily pain	85.7 (78.1–93.3)	77.6 (70.2–85.0)	0.11
	**Mental health**			
	Mental component summary	50.8 (47.4–54.2)	52.4 (49.1–55.8)	0.46
	Mental health	77.0 (71.1–83.0)	77.5 (71.7–83.4)	0.90
	Vitality	63.4 (56.7–70.1)	66.3 (59.7–72.9)	0.51
	Social functioning	91.3 (83.6–99.0)	92.4 (84.8–99.9)	0.83
	Role limitations due to emotional problems	89.1 (83.6–94.6)	93.2 (87.8–98.5)	0.26

## Discussion

We did not observe any associations between SES and HRQL in our group of overweight middle-aged men, whether assessed by NZDep2006 or capital value of residence. Our findings contrast to the results of Minet Kinge & Morris (2010) who observed lower HRQL scores among obese and overweight individuals with a lower SES than those of the same weight with a higher SES. Studies in Canada (Ross et al., 2012) and Greece (Pappa et al., 2009) also observed that greater affluence was associated with higher HRQL.

However, the existing evidence for the association between HRQL and SES is not consistent. In Japan, Yamazaki, Fukuhara & Suzukamo (2005) observed a strong association between income and HRQL amongst Japanese men but not women, while an older study in that country found a very modest effect of income on HRQL (Asada & Ohkusa, 2004). Pappa et al. (2015) observed that among Roma adults in Greece material deprivation was associated with lower HRQL scores on certain domains (general health and vitality), but with higher HRQL scores on others (role physical and role emotional), findings that the authors were unable to explain. Other studies focusing on physical health (more specifically on prevalence and characteristics of the metabolic syndrome) also failed to observe any association with HRQL among men in the United States (Chichlowska et al., 2008) and South Korea (Lim et al., 2012). It is possible that some of these inconsistencies may be associated with for example, varying levels of education, as previous studies have shown a strong relationship between education levels and HRQL (Devlin, Hansen & Herbison, 2000; Lacey & Walters, 2003; Mielck, Vogelmann & Leidl, 2014). Of note, Miravitlles et al. (2011) observed that lower levels of education were associated with lower HRQL, but no association was observed for occupational status. Thus, it is possible that while we did not observe an association between area-level deprivation or capital value of residence with HRQL, the results might have been different if it had been possible to account for the participants’ levels of education.

Nonetheless, while SES did not appear to affect HRQL in our participants at the time of assessment, it is unlikely to remain unimportant over time. It has been proposed that people of lower SES face a ’double burden’, namely increased health complications as well as lower HRQL when their health declines (Mielck, Vogelmann & Leidl, 2014). Participants in our study were middle-aged, overweight but not obese, did not smoke, and had no significant comorbid conditions. Over time, these men may put on more weight, and adult weight gain is associated with increased risk of chronic diseases in men (De Mutsert et al., 2014). This progression from overweight to a diseased state is more likely to occur in those of lower SES. Thus, even though our results covered a period of approximately 30 weeks, our findings may not reflect the trend over years, and over time it is likely that those of lower SES would have a larger reduction in HRQL than those who are wealthier.

Lower SES is associated with increased morbidity and mortality. A recent review of the association between SES and cardiovascular disease and/or cardiovascular risk factors, reported a clear inverse gradient in high income countries (De Mestral & Stringhini, 2017). Several studies have also identified a link between SES and type 2 diabetes (Bird et al., 2015; Espelt et al., 2013; Hwang & Shon, 2014). Early identification of modifiable risk factors and appropriate lifestyle changes can improve health outcomes and reduce the onset of chronic disease. However, if the health of at-risk middle-age men is not affecting their quality of life, they are less likely to meet a health professional. Health promotion efforts are also more likely to be acted upon by those of higher SES (Adler & Newman, 2002). Thus, our findings are of relevance as they underscore the importance of health promotion that focuses on disease prevention and identification of risk factors in men who *feel well*, particularly targeted to those of low SES.

The main limitations of our study were the secondary analyses of our clinical trial data, our relatively small number of participants (*n* = 38), and a selected group of individuals (overweight males living in a large urban centre), which may limit wider applicability of our findings. Particularly, since our cohort was relatively narrow and recruited from a single centre in New Zealand. Further, as all participants were volunteers in a clinical trial examining the effects of a nutritional supplement on metabolism, they are potentially more motivated than equivalent groups in the wider population. We also acknowledge that other factors that have been shown to affect HRQL (such as the education levels of the individuals and their parents (Ross et al., 2012)) have not been controlled for, and could have affected study outcomes.

Nevertheless, our findings are likely to be robust as each participant underwent three assessments over a 30-week period, minimizing the potential effects of temporal variations in HRQL that individual participants might have experienced. In addition, the lack of associations in our study remained irrespective of whether SES was assessed using a measure of neighbourhood wealth (NZDep2006) or family wealth (capital value of residence). Lastly, we studied a relatively homogeneous group of men (mostly of New Zealand European ethnicity), which likely mitigated the confounding effects of ethnicity on study outcomes.

## Conclusion

Our findings show that SES per se was not associated with HRQL in middle-aged men who are overweight but otherwise healthy, an important and increasing group. While the equality of HRQL across the socioeconomic spectrum in this group would generally be considered favourable, overweight men of lower SES are known to have increased cardiometabolic risk. That this risk is not associated with impaired quality of life potentially reduces the likelihood that such men will to seek to improve their health. This finding underscores the fact that health promotion interventions aimed at improving the health of at-risk men should also aim to reach asymptomatic people who feel generally well.

##  Supplemental Information

10.7717/peerj.5193/supp-1 Data S1 Raw data

## Additional Information and Declarations

### Data Availability

The following information was supplied regarding data availability: The raw data are provided as a Supplemental File.

## References

[ref-1] Adler NE, Newman K (2002). Socioeconomic disparities in health: pathways and policies. Health Affairs.

[ref-2] Adler NE, Ostrove JM (1999). Socioeconomic status and health: what we know and what we don’t. Annals of the New York Academy of Sciences.

[ref-3] Alishiri GH, Bayat N, Fathi Ashtiani A, Tavallaii SA, Assari S, Moharamzad Y (2008). Logistic regression models for predicting physical and mental health-related quality of life in rheumatoid arthritis patients. Modern Rheumatology.

[ref-4] Asada Y, Ohkusa Y (2004). Analysis of health-related quality of life (HRQL), its distribution, and its distribution by income in Japan, 1989 and 1998. Social Science and Medicine.

[ref-5] Bird Y, Lemstra M, Rogers M, Moraros J (2015). The relationship between socioeconomic status/income and prevalence of diabetes and associated conditions: a cross-sectional population-based study in Saskatchewan, Canada. International Journal for Equity in Health.

[ref-6] Braveman P, Gottlieb L (2014). The social determinants of health: it’s time to consider the causes of the causes. Public Health Reports.

[ref-7] Chichlowska KL, Rose KM, Diez-Roux AV, Golden SH, McNeill AM, Heiss G (2008). Individual and neighborhood socioeconomic status characteristics and prevalence of metabolic syndrome: the Atherosclerosis Risk in Communities (ARIC) study. Psychosomatic Medicine.

[ref-8] De Bock M, Derraik JGB, Brennan CM, Biggs JB, Morgan PE, Hodgkinson SC, Hofman PL, Cutfield WS (2013). Olive (*Olea europaea* L.) leaf polyphenols improve insulin sensitivity in middle-aged overweight men: a randomized, placebo-controlled, crossover trial. PLOS ONE.

[ref-9] De Mestral C, Stringhini S (2017). Socioeconomic status and cardiovascular disease: an update. Current Cardiology Reports.

[ref-10] De Mutsert R, Sun Q, Willett WC, Hu FB, Van Dam RM (2014). Overweight in early adulthood, adult weight change, and risk of type 2 diabetes, cardiovascular diseases, and certain cancers in men: a cohort study. American Journal of Epidemiology.

[ref-11] Derraik JGB, De Bock M, Hofman PL, Cutfield WS (2014). Increasing BMI is associated with a progressive reduction in physical quality of life among overweight middle-aged men. Scientific Reports.

[ref-12] Devlin N, Hansen P, Herbison P (2000). Variations in self-reported health status: results from a New Zealand survey. New Zealand Medical Journal.

[ref-13] Espelt A, Borrell C, Palència L, Goday A, Spadea T, Gnavi R, Font-Ribera L, Kunst AE (2013). Socioeconomic inequalities in the incidence and prevalence of type 2 diabetes mellitus in Europe. Gaceta Sanitaria.

[ref-14] Ferrans CE, Zerwic JJ, Wilbur JE, Larson JL (2005). Conceptual model of health-related quality of life. Journal of Nursing Scholarship.

[ref-15] Frieling MA, Davis WR, Chiang G (2013). The SF-36v2 and SF-12v2 health surveys in New Zealand: norms, scoring coefficients and cross-country comparisons. Australian and New Zealand Journal of Public Health.

[ref-16] Hagstromer M, Oja P, Sjostrom M (2006). The International Physical Activity Questionnaire (IPAQ): a study of concurrent and construct validity. Public Health Nutrition.

[ref-17] Hwang J, Shon C (2014). Relationship between socioeconomic status and type 2 diabetes: results from Korea National Health and Nutrition Examination Survey (KNHANES) 2010–2012. BMJ Open.

[ref-18] Lacey EA, Walters SJ (2003). Continuing inequality: gender and social class influences on self perceived health after a heart attack. Journal of Epidemiology and Community Health.

[ref-19] Lim H, Nguyen T, Choue R, Wang Y (2012). Sociodemographic disparities in the composition of metabolic syndrome components among adults in South Korea. Diabetes Care.

[ref-20] Loucks EB, Magnusson KT, Cook S, Rehkopf DH, Ford ES, Berkman LF (2007). Socioeconomic position and the metabolic syndrome in early, middle, and late life: evidence from NHANES 1999–2002. Annals of Epidemiology.

[ref-21] Mielck A, Vogelmann M, Leidl R (2014). Health-related quality of life and socioeconomic status: inequalities among adults with a chronic disease. Health and Quality of Life Outcomes.

[ref-22] Minet Kinge J, Morris S (2010). Socioeconomic variation in the impact of obesity on health-related quality of life. Social Science and Medicine.

[ref-23] Ministry of Health (2016). Annual update of key results 2015/16: New Zealand health survey.

[ref-24] Miravitlles M, Naberan K, Cantoni J, Azpeitia A (2011). Socioeconomic status and health-related quality of life of patients with chronic obstructive pulmonary disease. Respiration.

[ref-25] Osborn R, Squires D, Doty MM, Sarnak DO, Schneider EC (2016). In new survey of eleven countries, US adults still struggle with access to and affordability of health care. Health Affairs.

[ref-26] Ovayolu N, Ovayolu O, Karadag G (2011). Health-related quality of life in ankylosing spondylitis, fibromyalgia syndrome, and rheumatoid arthritis: a comparison with a selected sample of healthy individuals. Clinical Rheumatology.

[ref-27] Pappa E, Chatzikonstantinidou S, Chalkiopoulos G, Papadopoulos A, Niakas D (2015). Health-related quality of life of the Roma in Greece: the role of socio-economic characteristics and housing conditions. International Journal of Environmental Research and Public Health.

[ref-28] Pappa E, Kontodimopoulos N, Papadopoulos AA, Niakas D (2009). Assessing the socio-economic and demographic impact on health-related quality of life: evidence from Greece. International Journal of Public Health.

[ref-29] Robert SA, Cherepanov D, Palta M, Dunham NC, Feeny D, Fryback DG (2009). Socioeconomic status and age variations in health-related quality of life: results from the national health measurement study. Journals of Gerontology, Series B: Psychological Sciences and Social Sciences.

[ref-30] Ross NA, Garner R, Bernier J, Feeny DH, Kaplan MS, McFarland B, Orpana HM, Oderkirk J (2012). Trajectories of health-related quality of life by socio-economic status in a nationally representative Canadian cohort. Journal of Epidemiology and Community Health.

[ref-31] Salmond C, Crampton P (2012). Measuring socioeconomic position in New Zealand. Journal of Primary Health Care.

[ref-32] Salmond C, Crampton P, Atkinson J (2007). NZDep 2006 index of deprivation.

[ref-33] Ware Jr JE (2000). SF-36 health survey update. Spine.

[ref-34] Yamazaki S, Fukuhara S, Suzukamo Y (2005). Household income is strongly associated with health-related quality of life among Japanese men but not women. Public Health.

